# *Mycoplasma genitalium* among Young, Urban Pregnant Women

**DOI:** 10.1155/2010/984760

**Published:** 2010-03-31

**Authors:** Vanessa L. Short, Jørgen S. Jensen, Deborah B. Nelson, Pamela J. Murray, Roberta B. Ness, Catherine L. Haggerty

**Affiliations:** ^1^Department of Epidemiology, Graduate School of Public Health, University of Pittsburgh, Pittsburgh, PA 15261, USA; ^2^Mycoplasma Laboratory, Statens Serum Institut, DK-2300 Copenhagen, Denmark; ^3^Department of Public Health, Temple University, Philadelphia, PA 19122, USA; ^4^Department of Pediatrics, University of Pittsburgh School of Medicine, Pittsburgh, PA 15261, USA; ^5^The University of Texas School of Public Health, Houston, TX 77225, USA

## Abstract

*Objective*. As the consequences of *Mycoplasma genitalium* in pregnant women are unknown, we examined the relationship between prenatal *M. genitalium* infection and SAB. *Methods*. The presence of *M. genitalium* was determined by PCR in urine from 82 women who subsequently experienced a SAB and 134 women who maintained their pregnancies past 22 weeks gestation. The relationships between *M. genitalium* and subsequent SAB, demographic, current pregnancy, and reproductive health history characteristics were evaluated. *Results*. Compared to women without *M. genitalium*, women with *M. genitalium* were more likely to report nulliparity (41.7% versus 17.4%, *P* = .04), history of pelvic inflammatory disease (27.3% versus 8.8%, *P* = .08), prior *C. trachomatis* infection (63.6% versus 36.9%, *P* = .11,) and problems getting pregnant (18.2% versus 4.4%, *P* = .10). *M. genitalium* was not associated with SAB (AOR 0.9, 95% CI 0.2–3.8). *Conclusions*. Pregnant women who test positive for *M. genitalium* do not have an increased risk of SAB but report a history of reproductive morbidities.

## 1. Introduction

Spontaneous abortion (SAB), the loss of a conceptus prior to 20 weeks, is the most common adverse outcome of pregnancy, occurring in an estimated 15% of clinically recognized pregnancies [1] and up to 50% of all pregnancies [2]. Many SABs occurring in the first trimester are due to phenotypic and/or chromosomal abnormalities, while environmental factors may have a greater impact on SAB occurring in later pregnancy [2]. Evidence suggests that sexually transmitted bacterial and viral infections, such as *Treponema pallidum*, bacterial vaginosis (BV), and *Chlamydia trachomatis*, may play a role in SAB [3–13]. 

*Mycoplasma genitalium, *a sexually transmitted bacterium, has been linked to various adverse gynecologic and reproductive events. It has been associated with cervicitis [14–17] and has been implicated as an etiological agent of pelvic inflammatory disease (PID) independent of *C. trachomatis *and *Neisseria gonorrhoeae * [17–20]. Furthermore, it has been detected in cervical and salpingeal samples from women with laparoscopically confirmed salpingitis [21] and in cervical and endometrial specimens from women with histologically confirmed endometritis [18]. A serologic relationship between *M. genitalium* and tubal factor infertility has also been identified [22, 23].

Because *M. genitalium *has been associated with these morbidities, it is plausible that *M. genitalium *can infect the upper genital tract during pregnancy, resulting in adverse pregnancy outcomes. However, data on *M. genitalium* in pregnancy are sparse. Whereas two studies among low-risk women have reported no relationship between *M. genitalium* and SAB [11, 24], one study has reported an association between *M. genitalium* and preterm birth [25]. Thus, it is possible that *M. genitalium* may have an adverse effect on pregnancy outcomes. We conducted a nested case-control study to examine the role of *M. genitalium* in early pregnancy and subsequent SAB. The associations between *M. genitalium* and microbiologic, demographic, current pregnancy, and reproductive health history characteristics among predominantly young, single, African-American women with a history of sexually transmitted diseases (STDs) were also investigated.

## 2. Materials and Methods

### 2.1. Study Population

For our analyses, we used data from participants enrolled in the prospective Early Pregnancy Study (EPS), designed to examine the influence of violence on SAB risk [26]. During the period of January 1999 through August 2001, adolescent girls and women aged 14 to 40 years who presented to the emergency department (ED) of the hospital of the University of Pennsylvania with varying complaints, including pregnancy-related complications such as vaginal bleeding or abdominal pain, and nonpregnancy-related complaints related to infection, accidents, and injury, were screened for eligibility [26]. Nurse interviewers administered a brief screening questionnaire to identify all pregnant women less than 22 weeks gestation seen in the ED regardless of the reason for the visit. Among the 1,249 pregnant adolescent and adult women who were eligible for the study, 96% agreed to participate (*n* = 1,199). The mean gestational age at enrollment was 10.3 weeks. All participants provided written informed consent, and the protocol and consent forms were approved by the institutional review board of the University of Pennsylvania.

### 2.2. Data Collection

At enrollment, nurse interviewers administered an in-person questionnaire. The interview contained information regarding medical and reproductive history, sociodemographic factors, current level of social support, living arrangements, and complications of the pregnancy. Hair samples were collected to measure cocaine use, and mid-stream urine samples were collected to assess recent tobacco and alcohol use. Follow-up telephone interviews were conducted at 16 and 22 weeks of gestation to determine pregnancy status, and medical record reviews were conducted to confirm pregnancy outcome. The 807 women who remained pregnant through 22 weeks of gestation were classified as controls (67%), and the 392 women who experienced a noninduced pregnancy loss before 22 weeks of gestation were classified as cases (33%). Of the cases, 212 experienced a SAB at enrollment and 180 experienced a SAB during the follow-up period. 

For our subsequent EPS subanalyses, women who were experiencing a SAB at enrollment and women who did not have a urine sample available for analysis were excluded. Therefore, cases consisted of women who experienced a SAB during the follow-up period and had a frozen urine sample available for analysis (*n* = 95). We did a 2 : 1 match for controls and randomly selected women who did not experience a SAB and who had urine samples available for testing (*n* = 190). Demographic and patient characteristics between women with and without available samples did not differ significantly.

### 2.3. Detection of *M. genitalium* and *C. trachomatis*

In 2009, for this subsequent study, previously collected urine samples stored at −70^°  ^C were tested for *M. genitalium* and *C. trachomatis*. *M. genitalium *PCR testing has been conducted in other studies stored frozen specimens for up to eight years [19, 27, 28]. Of the 285 samples sent for analysis, 216 samples (82 cases and 134 controls) were sufficient for analysis. *M. genitalium* was detected by an inhibitor-controlled TaqMan real-time PCR detecting a conserved part of the MgPa adhesion gene (MG192) [29]. All positive results were confirmed by a second PCR with primers deduced from the 16 S rRNA gene sequence of *M. genitalium* [29]. *C. trachomatis* DNA was detected by an inhibitor-controlled TaqMan real-time PCR amplifying a sequence of the 16S rRNA gene of chlamydia species with a *C. trachomatis* specific TaqMan MGB probe [29]. Positive results were confirmed by a real-time PCR assay detecting a sequence of the cryptic plasmid. The methods for PCR detection of *M. genitalium* and *C. trachomatis* in urine have been previously validated [30].

### 2.4. Microbiologic, Demographic, Current Pregnancy, and Reproductive Health History Variables

The following is a list of microbiologic, demographic, current pregnancy, and reproductive health history variables that were compared between cases and controls: *M. genitalium* and *C. trachomatis *infection detected by PCR, age, race, level of education, marital status, recipient of government assistance, gestational age at enrollment, vaginal bleeding during pregnancy, parity, history of STDs, history of PID, history of problems getting pregnant, history of SAB, prior ectopic pregnancies, and alcohol, cigarette, marijuana, and crack/cocaine use during current pregnancy. All variables were self-reported during the baseline interview. Cocaine use was additionally measured in hair specimens as previously described [26]. For our analysis, we defined cocaine use as cocaine detected in hair specimen collected at baseline and/or self-reported cocaine use. Gestational age was calculated on the basis of self-reported date of the last menstrual period. 

### 2.5. Statistical Methods

The microbiologic, demographic, current pregnancy, and reproductive health history characteristics among cases and controls were compared using *t*, chi-square, and Fisher's exact tests for continuous and dichotomous variables as appropriate. Associations between *M. genitalium* PCR and microbiologic, demographic, current pregnancy, and reproductive health history characteristics were assessed with univariate logistic regression models. Variables significant in the univariate model (*P*-value ≤ .10) were adjusted for *C. trachomatis* PCR in multivariate logistic regression models. Nulliparity was further adjusted for age. Fisher's exact test was used to test for associations if there were cell sizes of less than five in the contingency table.

Logistic regressions were used to investigate the relationship between *M. genitalium* urine PCR and SAB. We had a power of 80% to detect a 3.4 increased risk of SAB among women infected with *M. genitalium*. Analyses were repeated controlling for age, history of SAB, smoking, and gestational age. Regression models were repeated without history of SAB, as including the history of SAB variable in the model may bias the relationship toward the null if there is an association between *M. genitalium* and SAB. We repeated our analyses categorizing SAB by gestational age (<11 weeks, ≥11 weeks), since gestational age of 11 weeks or greater at time of SAB can be used as a marker for when pregnancies are more likely to be chromosomally normal and other factors such as infectious agents are more likely to cause pregnancy loss [2]. We also repeated our analyses excluding women who experienced vaginal bleeding at enrollment, as these women may have already been experiencing a SAB and this may have compromised temporality.

## 3. Results

Among all study participants, 5.6% (12/216) tested positive for *M. genitalium *and 6.9% (15/216) tested positive for *C. trachomatis *at enrollment. Only one woman was coinfected with both bacteria. Participants were predominantly less than 30 years old (82%), African-American (90%), not married (81%), and receiving government assistance at time of enrollment (59%). Nearly two-thirds of the women (62%) reported a history of STDs. The mean gestational age at enrollment was 9.2 weeks. 

Compared to control women, cases were significantly more likely to report vaginal bleeding both within 24 hours of the enrollment visit (57.3% versus 21.8%, *P* < .0001) and at any time during pregnancy (65.4% versus 37.3%, *P* < .0001) (Table 1). Cases were also significantly more likely to report a previous SAB (40.2% versus 26.1%, *P* = .03) than controls. These were the only characteristics that differed between cases and controls. Results remained the same after excluding women with vaginal bleeding at baseline.

Compared to women who tested negative for *M. genitalium*, women who tested positive for *M. genitalium* were more likely to report nulliparity (41.7% versus 17.4%, *P* = .04), a history of PID (27.3% versus 8.8%, *P* = .08), prior *C. trachomatis* infection (63.6% versus 36.9%, *P* = .11), and problems getting pregnant (18.2% versus 4.4%, *P* = .10) (Table 2). Although results were not statistically significant, the direction of the association remained the same after adjusting for *C. trachomatis* PCR (nulliparity AOR 3.4, 95% CI 1.0–11.6; history of PID AOR 3.9, 95% CI 0.9–16.1; prior *C. trachomatis *infection AOR 3.0, 95% CI 0.8–10.5; problems getting pregnant AOR 4.8, 95% CI 0.9–25.7). After adjusting for age, nulliparous women were still more likely to test positive for *M. genitalium *than women who did not report nulliparity, but the results were not statistically significant (AOR 2.8, 95% CI 0.8–9.8). 

*M. genitalium* was not independently associated with SAB, after adjusting for age, history of SAB, smoking during pregnancy, and gestational age (AOR 0.9, 95% CI 0.2–3.8) (Table 3). Results were similar after further adjusting for self-reported BV (AOR 0.4, 95% CI 0.08–1.9) and after excluding women who experienced vaginal bleeding at enrollment (OR 0.4, 95% CI 0.1–3.8). When history of SAB was removed from the multivariate model, *M. genitalium *was not associated with SAB.

Nearly 28% (22/79) of the cases experienced a SAB at less than 11 weeks gestation. Among these cases, none tested positive for *M. genitalium*. Among the cases that experienced a SAB at 11 or greater weeks of gestation, 4.3% (2/46) tested positive for *M. genitalium*. *M. genitalium* was not associated with SAB (OR 0.6, 95% CI 0.1–3.0), even after adjusting for age, history of SAB, smoking and gestational age at enrollment (AOR 1.0, 95% CI 0.2–6.5).

## 4. Disscusion

To our knowledge, ours was the first study to prospectively evaluate the relationship between *M. genitalium* and subsequent SAB among a high-risk pregnant population and we found that *M. genitalium* in pregnancy was not associated with subsequent SAB. Our null findings are consistent with those of two other studies of *M. genitalium* and SAB. Oakeshott et al. used PCR to test urine samples from 915 pregnant women presenting at prenatal care at less than 10 weeks gestation in general practice and family planning clinics in London [11]. Only 0.66% of the samples tested positive for *M. genitalium* [11]. Women who experienced a subsequent SAB were not more likely to test positive for *M. genitalium* compared to the women who did not experience a SAB (1% versus 0.6%, *P* = *N* *S*) [11]. Although the rate of *M. genitalium* was higher among SAB cases in this study, the power to detect a significant difference was likely limited, due to the small number of women testing positive for *M. genitalium *(*n* = 6) [11]. In a case-control study of 1014 recently pregnant women in Guinea-Bissau, *M. genitalium* detected by PCR in cervical samples collected within 24 hours of a SAB or delivery of a term neonate was not associated with SAB (OR 0.61, 95% CI 0.07–2.5) [24]. In our study, although we had a modest sample size, *M. genitalium* was more prevalent than reported in the study by Oakeshott et al. (5.6% versus 0.66%), and we had a power of 80% to detect approximately a threefold increased risk of SAB among *M. genitalium* positive patients. Our higher prevalence might be expected, as women in our study population were probably at high risk for STDs in general, as they were predominantly young, single, African-American, of lower socioeconomic status (SES) and reported a history of STDs. Further, in contrast to Labbe et al., who tested for *M. genitalium* at the time of SAB and delivery, we tested for *M. genitalium* prior to pregnancy loss. Therefore, unlike the previous studies of *M. genitalium* and SAB, our prospectively designed study allowed us to establish a temporal relationship between early pregnancy *M. genitalium* and subsequent SAB among a high-risk pregnant population.

In our study, the clinical, demographic, and behavioral characteristics of pregnant women who tested positive for *M. genitalium* and women who did not were similar. Our results are similar to those reported by Labbe et al., in which *M. genitalium *was not associated with any demographic, behavioral, clinical, or laboratory characteristic, including age, age at sexual debut, number of sexual partners, previous pregnancies, syphilis, HIV serology, *N. gonorrhoeae* and *T. vaginalis *(*P* > .05 for all) [24]. Conversely, Oakeshott et al. found that *M. genitalium* was more common in pregnant women who were less than 20 years of age (*P* = .002), black (*P* = .034), single (*P* = .017), and had lower SES (*P* = .007) [11]. Our null findings were likely due to the fact that our study population was a fairly homogeneous group of women, which may have biased our results toward the null.

Although *M. genitalium* was not associated with SAB or demographic and behavioral characteristics, it was associated with a history of other gynecologic and reproductive morbidities, including history of PID, prior chlamydial infection and problems getting pregnant. Further, our results suggest an independent relationship between *M. genitalium* and these conditions, as the results remained the same even after adjusting for the presence of *C. trachomatis*. Our results are consistent with other studies that have identified *M. genitalium* as a possible etiologic agent of other adverse reproductive conditions, such as nongonococcal/nonchlamydial PID [17–20] and PID-associated sequelae [19], including tubal factor infertility [22, 23]. It may be that *M. genitalium* was not associated with SAB in our study population of pregnant women because women with *M. genitalium* go on to develop PID and subfertility and would therefore not be enrolled in our study. Results from this study support the need for further research on the role of *M. genitalium* in reproductive and adverse pregnancy outcomes. While it is suggested that *M. genitalium* is associated with impaired fertility, larger, prospective studies among pregnant women and women trying to conceive are needed.

 There are a few limitations to recognize as part of this study. First, the small sample sizes of some of our subgroups limited statistical power. Second, the mean gestational age at enrollment was approximately 9 weeks, so earlier SABs would have been missed. Still, the gestational age ranged from 1 to 15, so early SAB was included. Furthermore, in subgroup analyses stratified by gestational age at enrollment, results were not different. Third, the use of self-report as a means of collecting information can introduce several biases and may not be a very accurate measure. Although gestational age was calculated on the basis of self-reported date of the last menstrual period, which may over or underestimate the true date, nearly 72% of participants had their estimated due date confirmed by pelvic ultrasound. Finally, only archived mid-stream urine (MSU) samples were available for *M. genitalium* testing in our EPS substudy, and this may have limited our analyses. While there are no data on the sensitivity of *M. genitalium* PCR in MSU versus first void urine (FVU) samples, *C. trachomatis* studies have found that MSU had a sensitivity of 86% to 95% compared to FVU [31]. Thus, we expect that MSU *M. genitalium* PCR yielded results in our study which would be similar to those using FVU, which Jensen et al. have previously published as the most sensitive specimen type for the detection of *M. genitalium *by PCR, as compared to cervical and urethral swab specimens [30]. In a study comparing the efficacy of FVU with cervical and urethral swab specimens for detection of *M. genitalium *using in-house inhibitor-controlled PCR assays, more infections were detected using urine samples (88%) than cervical (71%) and urethral swab specimens (57%), and the urine specimen was significantly more efficient than both the cervical (*P* = .049) and the urethral swab specimen (*P* = .0009) [30]. However, for optimal sensitivity, urine should be supplemented with a cervical specimen. In our study, the addition of a cervical or vaginal specimen for PCR may have increased the sensitivity and detected more *M. genitalium* positive women. However, cervical samples were not available for testing. Therefore, the reported prevalence of *M. genitalium* in our study population may be an underestimate of the true prevalence in our study population. The null relationship between *M. genitalium* urine PCR and SAB may further be explained by the fact that some women did not have upper genital tract infection, but instead had only urinary or lower genital tract infection, insufficient to cause SAB. 

## 5. Conclusions

In conclusion, among this population of mostly young, single, African-American pregnant women, *M. genitalium* was not predictive of SAB. However, trends between *M. genitalium *and a history of gynecologic and reproductive morbidities, including other STDs, PID, and problems getting pregnant were evident, even after adjusting for *C. trachomatis*. Results from this study support the need for further research on the role of *M. genitalium* in reproductive and adverse pregnancy outcomes. While it is suggested that *M. genitalium* is associated with impaired fertility, larger, prospective studies among pregnant women and women trying to conceive are needed. Such studies should consider testing for *M. genitalium* in cervical or vaginal specimens from women recruited prior to pregnancy or from women early in gestation.

## Figures and Tables

**Table 1:** Characteristics of women who experienced a SAB and women who maintained their pregnancies past 22 weeks gestation.

Characteristic	Women who experienced a SAB *n* = 82	Women who maintained their pregnancies *n* = 134	*P*-value
	*n* (%)	*n* (%)	
Infection			

* M. genitalium*	3 (3.7)	9 (6.7)	.54
* C. trachomatis*	3 (3.7)	12 (9.0)	.17
* M. genitalium *and* C. trachomatis *	0	1 (0.7)	1.00

Demographic			

Mean age (standard deviation)	24.8 (6.2)	23.9 (5.7)	.33
Age			
< 25 years	49 (59.7)	92 (68.7)	.18
≥ 25 years	33 (40.2)	42 (31.3)	
Race/ethnicity			
African-American	72 (88.9)	120 (89.6)	.89
Other	9 (11.1)	14 (10.4)	
Marital Status			
Unmarried	68 (82.9)	107 (79.8)	.57
Married	14 (17.1)	27 (20.2)	
Education			
< High school	25 (30.5)	50 (37.3)	.31
≥ High school	57 (69.5)	84 (62.7)	
Receiving government assistance	31 (37.8)	58 (43.3)	.43

Current pregnancy history			

Mean gestational age^(a)^ (standard deviation)	8.7 (3.2)	9.5 (3.4)	.08
Vaginal bleeding at enrollment	47 (57.3)	29 (21.8)	<.0001
Vaginal bleeding since LMP	53 (65.4)	50 (37.3)	<.0001
Alcohol use	30 (36.6)	44 (32.8)	.57
Cigarette smoking	31 (38.3)	44 (32.8)	.42
Marijuana use	17 (21.0)	25 (18.7)	.68
Crack/cocaine use^(b)^	16 (25.4)	21 (22.8)	.71

Reproductive health history			

Nulliparous	14 (17.1)	27 (20.1)	.58
Prior SAB	34 (40.2)	35 (26.1)	.03
Prior ectopic pregnancy	5 (7.3)	6 (5.6)	.75
History of STD^(c)^	48 (60.0)	82 (62.1)	.76
History of PID	6 (7.4)	15 (11.3)	.36
History of problems getting pregnant	5 (6.3)	6 (4.5)	.75

Note: Missing observations: race, *n* = 1; vaginal bleeding, *n* = 1; cigarette smoking, *n* = 1; marijuana use, *n* = 1; crack/cocaine use, *n* = 61; prior incompetent cervix, *n* = 2; prior ectopic pregnancy, *n* = 41; history of STD, *n* = 5; history of PID, *n* = 3; history of problems getting pregnant, *n* = 2.

^(a)^Gestational age at enrollment in weeks.

^(b)^Detected in hair sample collected at enrollment and/or self-report cocaine use.

^(c)^ *C. trachomatis*, *N. gonorrhoeae*, syphilis, genital warts, *Trichomonas vaginalis*, and/or BV.

**Table 2:** Characteristics of study participants according to *M. genitalium* PCR result.

Characteristic	*M. genitalium* positive *n* = 12	*M. genitalium* negative *n* = 207	*P*-value
	*n* (%)	*n* (%)	
Coinfection			

*C. trachomatis*			
Yes	1 (8.3)	14 (6.8)	.83
No	11 (91.7)	193 (93.2)	

Demographic			

Age			
< 25 years	10 (83.3)	133 (64.2)	.22
≥ 25 years	2 (16.7)	74 (35.8)	
Race/ethnicity			
African American	11 (91.7)	184 (89.3)	.80
White/Other	1 (8.3)	22 (10.7)	
Marital status			
Unmarried	12 (100)	166 (80.2)	—^(a)^
Married	0	41 (19.8)	
Education			
< High school	5 (41.7)	72 (34.8)	.63
≥ High school	7 (58.3)	135 (65.2)	

Current pregnancy history			

Mean gestational age^(b)^ (standard deviation)	9.5 (2.9)	9.2 (3.3)	.78^(c)^
Vaginal bleeding at enrollment			
Yes	4 (33.3)	74 (35.9)	1.00
No	8 (66.7)	132 (64.1)	
Vaginal bleeding since LMP			
Yes	5 (41.7)	101 (49.0)	.62
No	7 (58.3)	105 (51.0)	
Alcohol use			
Yes	5 (41.7)	70 (33.8)	.87
No	7 (58.3)	137 (66.2)	
Cigarette use			
Yes	6 (50.0)	71/206 (34.5)	.28
No	6 (50.0)	135/206 (65.5)	
Marijuana use			
Yes	4 (33.3)	40/206 (19.4)	.27
No	8 (66.7)	166/206 (80.6)	
Crack/cocaine use^(d)^			
Yes	2 (20.0)	36 (24.3)	.75
No	8 (80.0)	112 (75.1)	

Reproductive health history			

Nulliparous			
Yes	5 (41.7)	36 (17.4)	.04
No	7 (58.3)	171 (82.6)	
History of SAB			
Yes	1 (8.3)	68 (32.8)	.07
No	11 (91.7)	139 (67.2)	
Prior ectopic pregnancy			
Yes	0 (0)	11 (6.4)	—^(a)^
No	7 (100)	160 (93.6)	
History of STD^(e)^			
Yes	11 (100)	121 (59.6)	—^(a)^
No	0 (0)	82 (40.4)	
History of *C. trachomatis *			
Yes	7 (63.6)	76 (36.9)	.11
No	4 (36.4)	130 (63.1)	
History of PID			
Yes	3 (27.3)	18 (8.8)	.08
No	7 (72.7)	187 (91.2)	
History of problems getting pregnant			
Yes	2 (18.2)	9 (4.4)	.10
No	9 (81.8)	197 (95.6)	

Note: Missing observations: race, *n* = 1; vaginal bleeding, *n* = 1; cigarette smoking, *n* = 1; marijuana use, *n* = 1; crack/cocaine use, *n* = 61; prior incompetent cervix, *n* = 2; prior ectopic pregnancy, *n* = 41; history of STD, *n* = 5; history of PID, *n* = 3; history of problems getting pregnant, *n* = 2.

^(a)^ *P*-value not calculated.

^(b)^Gestational age at enrollment in weeks.

^(c)^ *t*-test *P*-value = .78.

^(d)^Detected in hair sample collected at enrollment and/or self-reported cocaine use.

^(e)^ *C. trachomatis*, *N. gonorrhoeae*, syphilis, genital warts, *Trichomonas vaginalis*, and/or BV.

**Table 3:** Odds of spontaneous abortion according to infection at enrollment.

Infection	Women who experienced a SAB *n* = 82	Women who maintained their pregnancies *n* = 134	OR (95% CI)	AOR (95% CI)^(a)^
	*n* (%)	*n* (%)		
*M. genitalium*				
Yes	3 (3.7)	9 (6.7)	0.5 (0.1–2.0)	0.9 (0.2–3.8)
No	79 (96.3)	125 (93.3)		

^(a)^Adjusted for age, history of SAB, smoking, and gestational age.
